# Systematic Review and Meta-Analysis of Doxycycline Efficacy for Rectal Lymphogranuloma Venereum in Men Who Have Sex with Men

**DOI:** 10.3201/eid2210.160986

**Published:** 2016-10

**Authors:** Charussri Leeyaphan, Jason J. Ong, Eric P.F. Chow, Fabian Y.S. Kong, Jane S. Hocking, Melanie Bissessor, Christopher K. Fairley, Marcus Chen

**Affiliations:** Mahidol University, Bangkok, Thailand (C. Leeyaphan);; Alfred Health, Melbourne, Victoria, Australia (C. Leeyaphan, J.J. Ong, E.P.F. Chow, M. Bissessor, C.K. Fairley, M. Chen);; Monash University, Melbourne (J.J. Ong, E.P.F. Chow, C.K. Fairley, M. Chen);; University of Melbourne, Melbourne (F.Y.S. Kong, J.S. Hocking)

**Keywords:** rectal lymphogranuloma venereum, treatment, chlamydia, Chlamydia trachomatis, doxycycline, men who have sex with men, systematic review, meta-analysis, efficacy, sexually transmitted infection, STI, bacteria

## Abstract

Rectal lymphogranuloma venereum (LGV) has reemerged as a sexually transmitted infection among men who have sex with men (MSM), particularly those who are HIV-positive. We undertook a systematic review and meta-analysis to determine the efficacy of doxycycline (100 mg 2×/d for 21 days) for rectal LGV in MSM. Nine studies were included: 4 prospective, 4 retrospective, and 1 combined retrospective and prospective. In total, 282 MSM with rectal LGV were included in the studies. All studies reported using nucleic acid amplification tests to assess microbial cure. Most patients (>80%) had symptomatic rectal infection. The fixed-effects pooled efficacy for doxycycline was 98.5% (95% CI 96.3%–100%, *I*^2^ = 0%; p = 0.993). Doxycycline at 100 mg twice daily for 21 days demonstrated a high microbial cure rate. These data support doxycycline at this dosage and duration as first-line therapy for rectal LGV in MSM.

## Abstract 2

A high microbial cure rate was shown with 100 mg doxycycline twice daily for 21 days.

Lymphogranuloma venereum (LGV) has reemerged since the early 2000s as a cause of proctitis in men who have sex with men (MSM). Rectal LGV infections in MSM have been associated with high-risk sexual behaviors, increased rates of concurrent sexually transmitted infections (STIs), and hepatitis C, and the infections have been overrepresented among HIV-positive MSM (*1*). During 2003–2012, a total of 2,138 LGV cases were diagnosed in the United Kingdom: 98% were in MSM, of whom 80% were HIV-positive and 20% had hepatitis C infection (*2*). Surveys from Spain and Germany have shown that rectal LGV accounts for 8%–16% of rectal chlamydia infections in MSM (*3*,*4*).

LGV is caused by *Chlamydia trachomatis* serovars L1–L3 which, following mucosal inoculation, disseminate via underlying tissue to regional lymph nodes. This infection process contrasts with that of chlamydia infections caused by *C. trachomatis* serovars A–K, which are limited to the mucosa (*5*). Prior to the 21st century, LGV was endemic to Southeast Asia, the Caribbean, Latin America, and Africa, where infections mainly involved genital inoculation and ulceration with lymphatic spread, resulting in bubo formation (*5*,*6*). More recently, LGV infections among MSM have been largely attributable to the L2b variant of *C. trachomatis* and have predominantly presented as rectal infections following inoculation of the rectal mucosa (*3*). Compared with MSM who have rectal infections caused by chlamydia strains unrelated to LGV, MSM who have rectal LGV are more likely to have symptoms of proctitis and more likely to be HIV-positive (*7*). Symptomatic proctitis is a syndrome commonly seen among MSM attending STI clinics; cases caused by LGV and other sexually acquired pathogens are often clinically indistinguishable (*8*,*9*). Exudative proctitis has frequently been observed, by proctoscopy, in patients with rectal LGV (*10*,*11*). Asymptomatic rectal LGV also occurs and has accounted for different proportions of LGV cases in various studies (*7*,*12*,*13*).

A comparative study published in 1957 provided early evidence for the efficacy of tetracyclines for reducing bubo duration when used for 14 days (*14*). Several national and regional guidelines currently recommend doxycycline (100 mg 2×/d for 21 d) as first-line therapy for rectal LGV (*5*,*6*,*15*,*16*). The deep-seated nature of LGV infection is one rationale for this 3-week duration of treatment (*12*). In a prospective study using repeated testing to verify response to therapy, rectal chlamydia RNA was detectable for up to 16 days following commencement of doxycycline treatment, adding weight to the need for a longer course of doxycycline for LGV (*17*). However, several case reports have described doxycycline failing to cure LGV in MSM despite 21 days of therapy, including cases of LGV buboes and rectal LGV (*18*–*21*). To provide an evidence base for the use of doxycycline as treatment for rectal LGV infections in MSM, we conducted a systematic review and meta-analysis of studies reporting microbial cure among MSM with rectal LGV treated with 100 mg doxycycline twice daily for 21 days.

## Methods

### Protocol and Registration

This systematic review and meta-analysis was conducted and reported according to the PRISMA (Preferred Reporting Items for Systematic Reviews and Meta-Analyses) Statement (http://prisma-statement.org/). The study protocol was registered with PROSPRERO (International Prospective Register of Systematic Reviews, http://www.crd.york.ac.uk/PROSPERO/; registration no. CRD42016036038).

### Search Strategy

Six electronic bibliographic databases (Medline, Embase, PubMed, ClinicalTrials.gov, Cochrane Central Register of Controlled Trials, and the Australian New Zealand Clinical Trials Registry) were searched for studies from 1940 to February 2016. In addition, we hand-searched conference abstracts that were available from the International Society for Sexually Transmitted Diseases Research for 2003–2013 and from the International Union against Sexually Transmitted Infections for 2009–2015; we also searched the reference lists of identified papers. Only abstracts published in English were included in the review. No attempt was made to identify unpublished studies.

We used the following search terms: “lymphogranuloma venereum” or “LGV” or “lymphogranuloma venereum” and “treatment” or “LGV and treatment.” Medical subject headings (MeSH) were used where possible. To capture all relevant articles, we did not restrict the search strategy specifically to doxycycline or rectal LGV.

### Inclusion and Exclusion Criteria

We searched for any published studies providing data on microbial cure of rectal LGV in MSM. Patients were considered cured if a repeat anal swab sample was negative for* C. trachomatis* by nucleic acid amplification testing (NAAT) after treatment with 100 mg doxycycline twice daily for 21 days. So that study cure rates could be included in the meta-analysis, we required the following data for study inclusion: 1) the number of MSM with rectal LGV treated with 100 mg doxycycline twice daily for 21 days; 2) the number of these men who had repeat testing for rectal chlamydia infection following this treatment; and 3) the results of repeat testing for rectal chlamydia infection. Studies with bisexual men were included as studies with MSM. For studies in which the 3 data above were not clear from the published papers or abstracts, we contacted authors to directly request the information. If the information was obtained, the studies and their data were included in the meta-analysis. Studies were excluded if 1) cure rates for rectal infection specifically, as distinct from inguinal buboes, could not be obtained; 2) the total sample size of the study was <10; 3) infections were in heterosexual men only; or 4) if different drugs or dosing regimens were used. Conference abstracts were also included if they fulfilled the inclusion criteria.

### Data Extraction Process

We extracted the following data from each study: study design, treatment administered, sample size, proportion of rectal LGV infections that were symptomatic, the diagnostic method for assessing microbial cure, attrition rate of study subjects, and microbial cure after treatment. One author (C.L.) undertook selection of studies, and another (J.J.O.) checked the selection. Disagreements were resolved by discussion and consultation with a third author (M.C.) until a consensus was reached.

### Outcome

Treatment efficacy for doxycycline, as determined by microbial cure, was calculated by using the number of treated men with a negative repeat test result by NAAT as the numerator and the number of treated men who underwent repeat testing as the denominator. A single *C. trachomatis*–negative anal swab sample after treatment was considered confirmation of microbial cure. Likewise, a repeat anal swab test positive for a chlamydia strain genotype not associated with LGV was also considered confirmation of cure but was associated with chlamydia reinfection.

### Analysis

Meta-analysis was applied to calculate the pooled estimates of doxycycline efficacy. We used the *I*^2^ test to estimate the approximate proportion of variability in point estimates attributed to heterogeneity other than that due to chance (*22*). Random-effects model results were shown if *I*^2^ was >25%, and fixed-effects model results were shown if *I*^2^ was <25%.

### Assessment of Bias and Quality

Publication bias was not assessed using a funnel plot because <10 studies met the inclusion criteria (*23*). Two authors (C.L. and J.J.O.) independently assessed within-study bias using evaluation criteria reported elsewhere (*24*). Any discrepancies were resolved by recourse to a third author (M.C.). Meta-analysis was conducted using STATA version 13 (StataCorp LP, College Station, TX, USA).

## Results

### Study Selection and Characteristics

**Figure 1:**
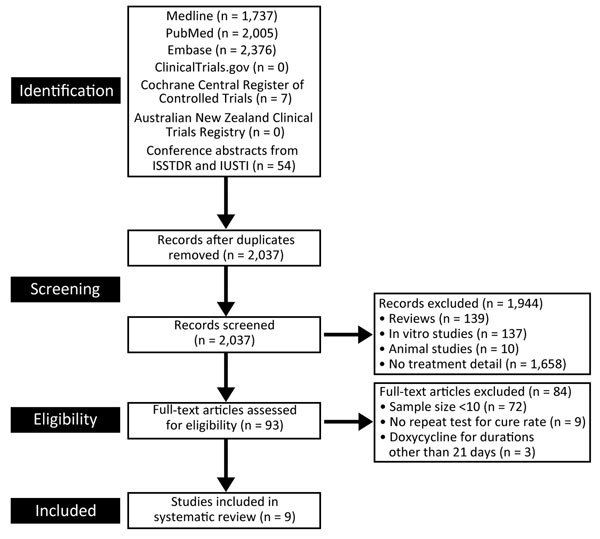
Studies reporting microbial cure after doxycycline treatment (100 mg 2×/d for 21 d) of rectal lymphogranuloma venereum in men who have sex with men. ISSTDR, International Society for Sexually Transmitted Diseases Research; IUSTI, International Union against Sexually Transmitted Infections.

We reviewed 93 of 2,037 identified studies; 9 met our inclusion criteria (Figure 1; Table 1). Five studies were presented in published articles (*3*,*17*,*25*–*27*), and 4 were presented as conference abstracts (*28*–*31*). For 6 of these 9 studies, we obtained additional data on rectal microbial cure rates through personal communications so that the studies could be included in our analysis (Table 1) (*3*,*26*,*28*–*31*). In 1 study, 100 mg doxycycline twice daily for 21 days was used as the comparator group; we used the microbial cure in the doxycycline group in our meta-analysis (*31*). Of the 9 studies, 4 were prospective (*3*,*17*,*27*,*31*), 4 were retrospective (*26*,*28*–*30*), and 1 was combined prospective and retrospective (*25*). All studies used NAAT for retesting after treatment. From these studies, data for a total of 282 MSM with rectal LGV who were retested after treatment with doxycycline were available and included in our meta-analysis. Eight studies reported that >80% of men had rectal symptoms when they sought medical care (*3*,*25*–*31*). All but 2 studies (*3*,*25*) reported the average time between treatment and repeat testing. A study by de Vries et al. (*17*) was the only study that undertook multiple repeat testing over time after doxycycline treatment; for the purposes of our analysis, we used the results from week 3 of the study because some men were given additional courses of doxycycline beyond 21 days. Five studies that reported attrition rates for repeat testing and the percentage of men who had repeat testing ranged from 0 to 65% (*17*,*26*,*27*,*30*,*31*).

**Table 1:** Studies from 2006 to 2015 reporting the efficacy of 100 mg doxycycline twice daily for 21 days for the treatment of rectal LGV in men who have sex with men*

Ref.	Study type	Serovar	No. men tested positive and treated for LGV/no. retested after treatment	No. symptomatic/no. total (%)	No. HIV-positive/no. total (%)	Method for retesting	Time from treatment to retesting	No. negative repeat test results/no. repeat tests (% negative; 95% CI)
(*25*)	RS/PS	L2	21/21	18/21 (86)	13/21 (62)	Cobas Amplicor Analyzer†	NS	21/21 (100; 85–100)
(*17*)	PS	L	20/17	NS	NS	Cobas Amplicor Analyzer†	3 wk	17/17 (100; 82–100)
(*26*)	RS	L	55/19	59/63 (94)	52/63 (82)	BD ProbeTec ET System‡	3 mo	19/19 (100; 83–100)§
(*28*)	RS	L	70/70¶	80/99 (81)	78/99 (79)	BD ProbeTec ET System‡	<6 mo	68/69 (99; 92–100)¶
(*29*)	RS	L2b	20/20#	20/20 (100)	18/25 (72)	BD ProbeTec ET System‡	3 mo	19/20 (95; 76–99)#
(*30*)	RS	L	80/43	71/83 (85)	69/83 (83)	APTIMA Combo 2 assay**	6 wk	42/43 (97; 88–100)††
(*27*)	PS	L2	13/13	13/13 (100)	9/13 (69)	Versant CT/GC DNA 1.0 assay‡‡	3 mo	13/13 (100; 77–100)
(*3*)	PS	L2, L2b	53/53§§	73/82 (89)	66/82 (80)	Abbott RealTime CT/NG assay¶¶, BD ProbeTec ET System‡	NS	51/53 (96; 87–99)§§
(*31*)	PS	L	28/27	28/28 (100)	27/28 (96)	Real-time multiplex PCR	3 wk	27/27 (100; 88–100)##

*LGV, lymphogranuloma venereum; NS, not specified; PS, prospective; ref., reference; RS, retrospective; RS/PS, retrospective and prospective. †Roche, Basel, Switzerland. ‡Becton Dickinson Microbiology Systems, Sparks, MD, USA. §S.C. Hill, St. Mary's Hospital, London, UK, pers. comm., 2016 Apr 28. ¶Seventy men treated for rectal LGV underwent repeat testing; 1 had an equivocal result and was excluded from further analyses (S. Pallawela, Royal Berkshire Hospital, Reading, UK, pers. comm., 2016 Apr 28). #M. Bissessor, Melbourne Sexual Health Centre, Carlton, Victoria, Australia, pers. comm., 2016 Apr 1. **Gen-Probe Inc., San Diego, CA, USA. ††A. Garner, Stockport NHS Foundation Trust, Stockport, UK, pers. comm., 2016 May 26. ‡‡Siemens Healthcare Diagnostics, Terrytown, NY, USA, §§J.C. Galan, Hospital Ramon y Cajal, Madrid, Spain, pers. comm., 2016 May 16. ¶¶Abbott Laboratories, Des Plaines, IL, USA ##J.L. Blanco, University of Barcelona, Barcelona, Spain, pers. comm., 2016 May 10.

### Treatment Efficacy

**Figure 2:**
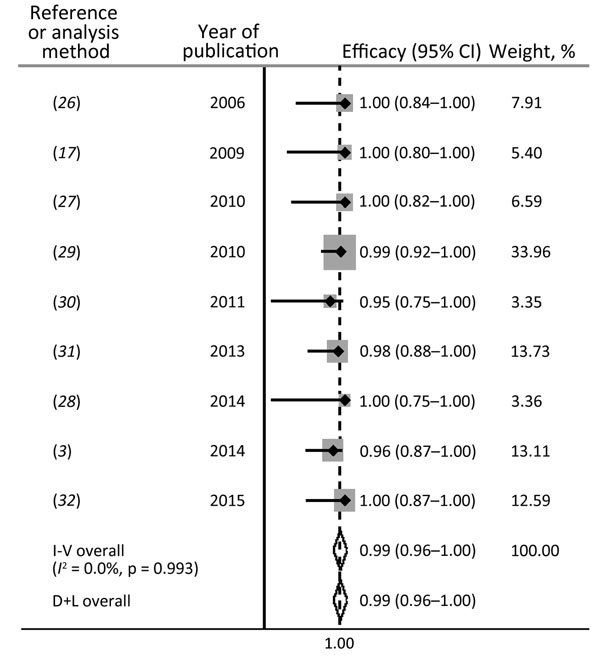
Efficacy of doxycycline (100 mg 2×/d for 21 d) for treatment of rectal lymphogranuloma venereum infection in men who have sex with men. I-V, inverse-variance (fixed) method; D+L, DerSimonian and Laird (random-effects) method; *I*^2^, test for heterogeneity.

The microbial cure rates for each of the 9 studies ranged from 95% (95% CI 76%–99%) (*29*) to 100% (95% CI 88%–100%) (*31*). Based on these 9 studies, the fixed-effects pooled efficacy was 98.5% (95% CI 96.3%–100%; *I*^2^ = 0%; p = 0.993) (Figure 2).

### Study Bias

We assessed the risk of biases within each of the 9 included studies (Table 2; Technical Appendix Table). Of the 9 studies, 7 recruited patients from STI clinics (*3*,*25*–*29*,*31*); 8 recruited MSM regardless of whether they had rectal symptoms (*3*,*17*,*25*–*28*,*30*,*31*); and 1 recruited only patients with symptomatic proctitis (*29*). All studies described in-house methods for LGV identification: 2 used a real-time PCR targeting the polymorphic membrane protein H gene (*17*,*26*); 1 used nested PCR and restriction fragment length polymorphism (RFLP) analysis targeting the major outer membrane protein gene (*25*); 1 used real-time multiplex PCR including LGV (*31*); 1 used PCR amplification of the outer membrane protein 1 (*omp*1) gene followed by RFLP analysis (*27*); 1 used *omp*1 gene sequencing (*29*); 1 used real-time polymorphic membrane protein H gene PCR and *omp*1 gene sequencing (*3*); 1 used LGV-specific molecular serovar typing (*28*); and 1 used an in-house assay (*30*).

**Table 2:** Summary of risk of bias in studies from 2006 to 2015 included in a systematic review and meta-analysis of the efficacy of doxycycline for rectal lymphogranuloma venereum in men who have sex with men*

Ref.	Method for selection of participants	Methods for measuring exposure and outcome variables	Design-specific sources of bias, excluding confounding	Method to control confounding	Statistical methods	Conflict of interest
(*25*)	+	+	NR	NR	+	NR
(*17*)	+	+	+	NR	+	+
(*26*)	+	+	++	NR	+	NR
(*28*)	+	+	++	NR	+	NR
(*29*)	+	+	++	NR	+	NR
(*30*)	+	+	++	NR	+	NR
(*27*)	+	+	++	NR	+	+
(*3*)	+	+	NR	NR	+	+
(*31*)	+	+	++	NR	+	NR

*NR, not reported; ref., reference; +, low risk of bias; ++, moderate risk of bias; +++, high risk of bias.

Studies differed in the manner in which they dealt with the possibility of LGV reinfection or non-LGV chlamydia as a cause of a positive test result after treatment. Three studies with participants whose repeat test results were positive reported that reinfection could have contributed to these results (*28*–*30*). Six of the studies conducted genotyping of positive repeat chlamydia specimens (*17*,*26*,*27*,*29*–*31*). Two of these studies reported genotyping results: Foschi et al*.* (*27*) reported that 1 patient had a non-LGV serovar on repeat testing, indicating chlamydia reinfection, and Bissessor et al. (*29*) demonstrated an LGV-associated serovar in a sample from a man who had unprotected sexual intercourse after treatment, making it impossible to determine whether the infection was due to treatment failure or reinfection. None of the studies reported the application of stringent criteria or algorithms, such as an algorithm based on a combination of sexual reexposure and chlamydia genotyping (*32*), to distinguish treatment failure from reinfection. All but 1 study based microbial cure on a single repeat test.

None of the studies discussed compliance with the full course of doxycycline or the concurrent use of other antimicrobial drugs, both of which could influence treatment outcomes. Three studies reported the authors’ conflicts of interest and funding sources (*3*,*17*,*27*). Through the review process, no randomized controlled trials comparing doxycycline with other treatments were identified.

## Discussion

Our meta-analysis found a pooled treatment efficacy of 98.5% (96.3%–100%; *I*^2^ = 0%) for 100 mg doxycycline twice daily for 21 days for the treatment of rectal LGV infections in MSM. This result supports the recommendation that doxycycline be used at this dosage and duration for the treatment of rectal LGV in MSM, including MSM who are HIV-positive. Several guidelines, including those from Europe, the United Kingdom, and the United States, recommend doxycycline at this dosage and duration as first-line therapy for the treatment of LGV (*5*,*6*,*15*). The results of our meta-analysis provide a high degree of precision to support the continuation of this recommendation.

Although rectal infections with LGV-associated variants of *C. trachomatis* have been concentrated among MSM, rectal chlamydia infections in MSM are still, overall, more likely to be caused by other chlamydia serovars. Infections with these other serovars have generally been treated with 100 mg doxycycline twice daily for 7 days, which has been shown from meta-analysis (*33*) to have a pooled efficacy of 99.6%. The efficacy of azithromycin (1 g) for rectal chlamydia infection has been questioned, and randomized trials comparing this with doxycycline for rectal chlamydia are needed. In a prospective study of rectal LGV treatment, 100 mg doxycycline twice daily for 7 days achieved a negative repeat chlamydia test result for only 15 (88%) of 17 MSM (*17*). Persistent rectal LGV infection despite 10 days of doxycycline treatment has also been reported (*18*). These reports underscore the value of genotyping positive rectal chlamydia specimens from MSM because the identification of LGV-associated variants will indicate the need for a longer course of doxycycline to ensure cure.

Although our meta-analysis showed that most rectal LGV infections were cured with a 21-day course of doxycycline, clinical failures have been reported with this regimen. In a case from France, an HIV-negative man with LGV proctitis and inguinal lymphadenopathy from the L2 serovar experienced clinical antimicrobial drug treatment failure even though doxycycline treatment was extended beyond 3 weeks. He subsequently achieved clinical resolution with moxifloxacin (*18*). In a report from Portugal, 2 HIV-positive MSM with rectal LGV from the L2b variant (1 with inguinal lymphadenopathy and the other with fever) did not clinically respond after 3 weeks’ treatment with doxycycline (*19*). In a case from Spain, a man with L2 serovar–associated LGV proctitis and inguinal lymphadenopathy received 200 mg doxycycline daily for 21 days, resulting in improved rectal symptoms, but progression of the lymphadenopathy required azithromycin followed by moxifloxacin to achieve clinical cure (*20*). There have also been reports of LGV buboes in MSM that have not resolved clinically after treatment with doxycycline for 21 days. In some cases, abscess formation and rupture with sinus formation have occurred despite this course of treatment (*21*,*34*). These reports suggest that in some cases of more clinically severe or extensive rectal LGV infection, 3 weeks’ treatment with doxycycline may not be sufficient for clinical and microbial cure. When abscesses are present, treatment failure might reflect poor penetration of antimicrobial drugs. Several national guidelines recommend that LGV infections be clinically observed until completely resolved and that routine test of cure is not necessary if a 21-day course of doxycycline has been completed (*5*,*6*,*15*). Our study findings support these recommendations.

Cases of rectal LGV among MSM have mainly been attributable to *C. trachomatis* serovars L2b and L2. Co-circulation of these 2 serovars among MSM in Spain has been shown with distinct clinical manifestations: LGV cases with rectal bleeding and pain have been associated with serovar L2b more than with L2 (*3*). Previous studies have, however, reported failure of doxycycline treatment in patients infected with the L2 serovar (*18*,*20*). Recently, a new LGV strain, L2c, a recombination of L2 and D strains, has been identified and may be associated with more severe infection (*35*). However, *C. trachomatis* serovar L, compared with serovars D–K, has no additional genes that determine disease outcome (*36*,*37*). Further research is required to define the determinants of LGV invasiveness and pathogenesis (*37*).

Several points should be considered when interpreting the results of our study. We identified only 9 studies that specifically reported microbial cure rates for MSM with rectal LGV, 4 of which were conference abstracts rather than published papers. The overall pooled efficacy of doxycycline was based on results for a total of 282 men. Although this combined number of cases is limited, the pooling together of otherwise small, individual studies provides a more precise estimate of doxycycline efficacy with tighter CIs than was previously available for each of the separate component studies (Table 1). Only 4 studies were prospective, and several were retrospective studies. We did not identify any randomized controlled trials. We identified considerable limitations in the quality of studies. The studies showed considerable variation in the timing of repeat testing after doxycycline, and only 1 study undertook repeat testing on more than 1 occasion (*17*). A single negative repeat test could, in theory, miss ongoing infection if organism shedding is intermittent and thus could lead to an overestimation of treatment efficacy (*38*). Conversely, a positive repeat test could indicate reinfection with LGV or another chlamydia strain rather than treatment failure, leading to an underestimation of treatment efficacy. None of the studies reported criteria or algorithms, as described previously (*32*), to distinguish treatment failure from reinfection based on sexual reexposure and chlamydia genotyping. Future randomized trials aimed at determining the efficacy of alternative antimicrobial drugs for the treatment of LGV are warranted and should include carefully considered algorithms that distinguish treatment failure from reinfection.

Despite these caveats, our study demonstrated a high microbial cure rate for 100 mg doxycycline twice daily for 21 days for the treatment of rectal LGV in MSM. The data support doxycycline at this dosage and duration as first-line therapy for rectal LGV.

Technical Appendix Risk bias assessment for studies included in a systematic review and meta-analysis of the efficacy of doxycycline for rectal lymphogranuloma venereum in men who have sex with men.
