# Supplementary material for: Systematic Review and Meta-Analysis of Doxycycline Efficacy for Rectal Lymphogranuloma Venereum in Men Who Have Sex with Men
Source: Emerg Infect Dis. 2016 Oct;22(10):1778–84. doi: 10.3201/eid2210.160986 (PMC5038401; doi:10.3201/eid2210.160986)
Supplement: Technical Appendix — Risk bias assessment for studies included in a systematic review and meta-analysis of the efficacy of doxycycline for rectal lymphogranuloma venereum in men who have sex with men. [file 16-0986-Techapp-s1.pdf]

Article DOI: http://dx.doi.org/10.3201/eid2210.160986

Systematic Review and Meta-Analysis of Doxycycline Efficacy for Rectal Lymphogranuloma Venereum in Men Who Have Sex with Men

Technical Appendix

Technical Appendix Table. Risk bias assessment for included studies.

Study, bias	Judgment	Support for judgment
**Waalboer R, 2009**		
Methods for selection of participants	Low risk	– **Setting where patients were recruited:** Reported<br>– Quote: "The study was conducted at the STI clinic of the Department of Dermatology and Venerology, Erasmus MC Rotterdam, Netherlands"<br>– **Selection of MSM with rectal LGV infection:** Reported<br>– Quote. "All patients attended the STI clinic on their own initiative because of symptoms related to STI or sexual risk behavior."<br>– Quote. "Rectal tests for gonococcal and chlamydial infection were performed as a routine in all MSM."<br>– Quote. "Genotyping was done prospectively in all rectal chlamydial infections …..February 2003. 33 (15.1%) rectal *Chlamydia trachomatis* infections from the years 2001 and 2002 were genotyped retrospectively."<br>– **Method for LGV identification:** Reported<br>– Quote. "Genotyping of the gene encoding the major outer membrane protein (MOMP) was performed by nested PCR and restriction fragment length polymorphism (RFLP) analysis"
Methods for measuring exposure and outcome variables	Low risk	– **Doxycycline 100 mg twice daily for 21 d was used as treatment:** Reported<br>– Quote. "….and (suspected) LGV infections with doxycycline 100 mg orally twice daily for 3 weeks"<br>– **Method used for repeat test after treatment:** Reported<br>– Quote. "….the automated *C. trachomatis* Cobas Amplicor PCR system was used throughout the study…"<br>– **Timing of the test of cure:** Not reported<br>– Quote. "Post-treatment swabs were only taken in those with LGV. All infections with LGV cleared after treatment with doxycycline."
Design-specific sources of bias (excluding confounding)	Not reported	– **Drug compliance:** Not reported<br>– **Sexual re-exposure as potential cause of repeat positivity:** Not reported<br>– **Genotype of positive repeat specimen for discrimination between reinfection and treatment failure:** Not reported<br>– **Antibiotic use shortly before doxycycline or before repeat testing:** Not reported
Methods to control confounding	Not reported	– Not reported
Statistical methods	Low risk	– Descriptive analysis used. No sample size calculation.

Study, bias	Judgment	Support for judgment
Conflict of interest	Not reported	– Not reported
**De Vries HJ, 2009**		
Methods for selection of participants	Low risk	– **Setting where patients were recruited:** Not reported<br>– **Selection of MSM with rectal LGV infection:** Reported<br>– Quote. "…,all men reporting receptive anal sex in the previous 6 months were routinely checked for chlamydia proctitis by collection of mucosal swab specimens during anoscopy"<br>– Quote. "Patients with chlamydia proctitis were asked at random for written consent to participate,… For all patients, biovar-L analysis of chlamydia-positive anal swab specimens was performed."<br>– Participants who missed >1 visit were excluded. Quote. "Thirty-one male patients with LGV proctitis and 31 with non-LGV chlamydia proctitis were included in the study. Eleven patients with LGV proctitis and 5 with non-LGV chlamydia proctitis missed >1 study visit and were excluded."<br>– **Method for LGV identification:** Reported.<br>– Quote. "….biovar-L analysis of chlamydia-positive anal swab specimens was performed, as described elsewhere [10, 11]."
Methods for measuring exposure and outcome variables	Low risk	– **Doxycycline 100 mg twice daily for 21 d was used as treatment:** Reported<br>– Quote. "If LGV proctitis was diagnosed (by biovar-L analysis) the doxycycline course was prolonged for 21 days."<br>– **Method used for repeat test after treatment:** Reported<br>– Quote. "…chlamydia DNA was detected by Cobas Amplicor (Roche, Basel, Switzerland)…"<br>– **Timing of the test of cure:** Reported.<br>– Quote. "Anal swab specimens were collected at subsequent visits during weeks 1, 2, 3, and 6 ...... after the commencement of doxycycline treatment)."
Design-specific sources of bias (excluding confounding)	Low risk	– **Repeated testing overtime after treatment conducted at week 1, 2, 3 and 6.**<br>– Quote. "Anal swab specimens were collected at subsequent visits during weeks 1, 2, 3, and 6 ...... after the commencement of doxycycline treatment)."<br>– **Drug compliance:** Not reported<br>– **Sexual re-exposure as potential cause of repeat positivity:** Reported<br>– Quote. "During this follow-up period, patients were asked to refrain from sexual contact."<br>– **Genotype of positive repeat specimen for discrimination between reinfection and treatment failure:** Reported<br>– Quote. "During this follow-up period….For chlamydia DNA–positive samples, biovar-L determination was performed"<br>– **Antibiotic use shortly before doxycycline or before repeat testing:** Reported<br>– There were additional treatments in 5 patients prior test of cure at 6 week.<br>– Quote. "At the end of therapy (week 3 visit), LGV proctitis persistent mucous membrane abnormalities were observed in 6 of 16 patients during anoscopic examination. For this reason, doxycycline treatment was prolonged for an additional 21 days for 5 of these 6 patients…all patients with LGV who had mucosal abnormalities after therapy completion showed microbial cure within the first week."
Methods to control confounding	Not reported	– Not reported
Statistical methods	Low risk	– Descriptive analysis used. No sample size calculation.
Conflict of interest	Reported conflict of interest	– Quote. "Financial support. Research and Development Fund of the Health Service Amsterdam. Potential conflicts of interest. All authors: no conflicts"
**Hill S, 2010**		
Methods for selection of participants	Low risk	– **Setting where patients were recruited:** Reported<br>– Quote. "The aim of this study was to audit the management of LGV within a large sexual health clinic in London, UK"<br>– **Selection of MSM with rectal LGV infection:** Reported

Study, bias	Judgment	Support for judgment
		– Retrospective study that reviewed all episodes of LGV. No criteria reported for patient selection for rectal chlamydia testing and genotyping.<br>– Quote. “We performed a retrospective case-note review of all patients with confirmed LGV between June 2005 and May 2009.”<br>– Quote. “Sixty-three episodes of LGV were diagnosed in 60 MSM…Three patients had two episodes of LGV during the audit period.”<br>– **Method for LGV identification:** Reported.<br>– Quote. “..real time PCR for the detection of LGV-specific DNA was performed…”
Methods for measuring exposure and outcome variables	Low risk	– **Doxycycline 100 mg twice daily for 21 d was used as treatment:** Reported<br>– Quote. “Fifty-five patients were treated with doxycycline in accordance with BASHH guidelines.”<br>– **Method used for repeat test after treatment:** Reported<br>– Quote. “Specimens were tested for CT using the BD ProbeTecTM ET System (Becton Dickinson Microbiology Systems, Sparks, MD, USA).”<br>– **Timing of the test of cure:** Reported.<br>– Quote. “…19 underwent TOC within three months of completing treatment.”
Design-specific sources of bias (excluding confounding)	Moderate risk	– **Drug compliance:** Not reported<br>– **Sexual re-exposure as potential cause of repeat positivity:** Not reported<br>– **Genotype of positive repeat specimen for discrimination between reinfection and treatment failure:** Reported<br>– Genotyping was undertaken in patients with positive rectal cases. Absence of LGV detection on repeat testing was classified as microbial cure in this study.<br>– Quote. “Twenty-one (87%) patients tested negative, and three (13%) tested positive for rectal CT – all with non-LGV strains”<br>– **Antibiotic use shortly before doxycycline or before repeat testing:** Not reported
Methods to control confounding	Not reported	– Not reported
Statistical methods	Low risk	– Descriptive analysis used. No sample size calculation.
Conflict of interest	Not reported	– Not reported
**Pallawela S, 2010**		
Methods for selection of participants	Low risk	– **Setting where patients were recruited:** Reported<br>– Quote. “A retrospective case note review was conducted in 6 GUM/HIV clinics in London.”<br>– **Selection of MSM with rectal LGV infection:** Reported<br>– Retrospective study reviewing all LGV cases with test of cure. Quote. “We identified 226 LGV MSM, of these 99 (44%) had a TOC within 6 months.<br>– From personal communication “There were 99 MSM with rectal LGV who underwent a repeat test included in this study.”<br>– **Method for LGV identification:** Reported<br>– Quote. “….LGV-specific molecular serovar typing”
Methods for measuring exposure and outcome variables	Low risk	– **Doxycycline 100 mg twice daily for 21 d was used as treatment:** Reported<br>– Quote. “The vast majority (94%) were treated with the recommended regimen of doxycycline”<br>– From personal communication “Of 99 MSM with rectal LGV, 70 patients received doxycycline 100 mg twice daily for 21 days.”<br>– **Method used for repeat test after treatment:** Reported<br>– Quote. “The standard test used for detecting *Chlamydia trachomatis* was the Becton Dickinson ProbeTecTM ET assay”<br>– **Timing of the test of cure:** Reported<br>– Quote. “….these 99 (44%) had a test of cure within 6 months….. The median time post treatment to test of cure was 68 days (range 17-185).”

Study, bias	Judgment	Support for judgment
Design-specific sources of bias (excluding confounding)	Moderate risk	– **Drug compliance:** Not reported<br>– **Sexual re-exposure as potential cause of repeat positivity:** Reported<br>– Quote. "One patient had an indeterminate test and one an equivocal result with high suspicion of re-infection: both tests were done before the recommended time."<br>– **Genotype of positive repeat specimen for discrimination between reinfection and treatment failure:** Not reported<br>– **Antibiotic use shortly before doxycycline or before repeat testing:** Not reported
Methods to control confounding	Not reported	– Not reported
Statistical methods	Low risk	– Descriptive analysis used. No sample size calculation.
Conflict of interest	Not reported	– Not reported
**Bissessor M, 2011**		
Methods for selection of participants	Low risk	– **Setting where patients were recruited:** Reported<br>– Quote. "We reviewed all cases of LGV seen at the Melbourne Sexual Health Centre between 2005 and 2010"<br>– **Selection of MSM with rectal LGV infection:** Reported<br>– **Method for LGV identification:** Reported.<br>– Quote. "During this period MSM who were diagnosed with rectal chlamydia using strand displacement assay were routinely questioned about symptoms of proctitis using a symptom check list. Those with symptoms of proctitis had specimens forwarded for *Chlamydia trachomatis omp*1 genotyping."
Methods for measuring exposure and outcome variables	Low risk	– **Doxycycline 100 mg twice daily for 21 d was used as treatment:** Reported<br>– Quote. "All but one of the LGV infected men received at least a 3-week course of doxycycline 100 mg twice daily." Personal communication "There were 20 men with rectal LGV who received doxycycline and had a repeat test."<br>– **Method used for repeat test after treatment:** Reported<br>– Quote. "…..diagnosed with rectal chlamydia using strand displacement assay"<br>– **Timing of the test of cure:** Reported for only cases with a positive repeat test.<br>– Quote. "Two men with ano-rectal LGV remained LGV positive on follow-up when tested 3 months later."
Design-specific sources of bias (excluding confounding)	Moderate risk	– **Drug compliance:** Not reported<br>– **Sexual re-exposure as potential cause of repeat positivity:** Reported<br>– Quote. "Both these patients were noted to have had unprotected sex following initial treatment, hence, it is unclear whether this was due to treatment failure or reinfection."<br>– **Genotype of positive repeat specimen for discrimination between reinfection and treatment failure:** Reported<br>– Quote. "Two men with ano-rectal LGV remained LGV positive on follow-up when tested 3 months later."<br>– **Antibiotic use shortly before doxycycline or before repeat testing:** Not reported
Methods to control confounding	Not reported	– Not reported
Statistical methods	Low risk	– Descriptive analysis used. No sample size calculation.
Conflict of interest	Not reported	– Not reported
**Vas A, 2013**		
Methods for selection of participants	Low risk	– **Setting where patients were recruited:** Reported<br>– Quote. "A three-centre retrospective cases note analysis" These three centers were Manchester Royal Infirmary; North Manchester General Hospital and Withington Community Hospital, Manchester, UK.<br>– **Selection of MSM with rectal LGV infection:** Reported<br>– Quote. "…83 patients diagnosed with LGV in Greater Manchester was performed. All patients were men and men who have sex with men (MSM),…" Personal communication "There were 43 men with rectal LGV who received doxycycline and had a repeat test in their clinic."<br>– **Method for LGV identification:** Not reported

Study, bias	Judgment	Support for judgment
Methods for measuring exposure and outcome variables	Low risk	– **Doxycycline 100 mg twice daily for 21 d was used as treatment:** Reported<br>– Quote. "All patients received first line treatment with a three-week course of doxycycline 100 mg twice daily orally."<br>– **Method used for repeat test after treatment:** Reported<br>– Personal communication "An in house assay was used for chlamydia and LGV detections."<br>– **Timing of the test of cure:** Reported<br>– Quote. "Test of cure at six weeks was documented in 52/83 cases and performed in 46 cases."
Design-specific sources of bias (excluding confounding)	Moderate risk	– **Drug compliance:** Not reported<br>– **Sexual re-exposure as potential cause of repeat positivity:** Reported<br>– Quote. "…one tested positive which was likely secondary to re-infection."<br>– **Genotype of positive repeat specimen for discrimination between reinfection and treatment failure:** Reported<br>– Personal communication "For test of cure, if chlamydia was positive, it was sent for LGV serovar testing."<br>– **Antibiotic use shortly before doxycycline or before repeat testing:** Not reported
Methods to control confounding	Not reported	– Not reported
Statistical methods	Low risk	– Descriptive analysis used. No sample size calculation.
Conflict of interest	Not reported	– Not reported
**Foschi C, 2014**		
Methods for selection of participants	Low risk	– **Setting where patients were recruited:** Reported<br>– Quote. "…patients attending the STI Outpatients Clinic of St. Orsola University Hospital of Bologna…"<br>– **Selection of MSM with rectal LGV infection:** Reported<br>– Quote. "From January 2012 to April 2013, all the patients attending…and reporting unsafe anal sexual intercourses have been asked to carry out a clinical examination. An anorectal swab, a pharyngeal swab (if reporting oral sex intercourses) and an urine sample were collected from each patient for DNA detection of CT and *Neisseria gonorrhoeae*."<br>– **Method for LGV identification:** Reported.<br>– Quote. "In case of a CT positive result, molecular genotyping, based on *omp1* gene semi-nested PCR, followed by RFLP analysis"
Methods for measuring exposure and outcome variables	Low risk	– **Doxycycline 100 mg twice daily for 21 d was used as treatment:** Reported<br>– Quote. "LGV positive patients were treated with doxycycline 100 mg twice a day for 3 weeks"<br>– **Method used for repeat test after treatment:** Reported<br>– Quote. "…anorectal and pharyngeal swabs were processed by Versant CT/GC DNA 1.0 Assay (Siemens Healthcare Diagnostics, Terrytown, NY, USA), a Real-Time PCR test simultaneously detecting the presence of CT and/or GC DNA"<br>– **Timing of the test of cure:** Reported<br>– Quote. "Three months after treatment, rectal swabs and urine samples were negative both for CT and GC nucleic acids in all patients but one."
Design-specific sources of bias (excluding confounding)	Moderate risk	– **Drug compliance:** Not reported<br>– **Sexual re-exposure as potential cause of repeat positivity:** Not reported<br>– **Genotype of positive repeat specimen for discrimination between reinfection and treatment failure:** Reported<br>– Quote. "This patient had a positive result for GC and a non-LGV serovar CT in his rectal swab, with recurrence of mild anal symptoms."<br>– **Antibiotic use shortly before doxycycline or before repeat testing:** Not reported
Methods to control confounding	Not reported	– Not reported
Statistical methods	Low risk	– Descriptive analysis used. No sample size calculation.
Conflict of interest	Reported conflict of	– Quote. "The authors declare that they have no competing interests."

Study, bias	Judgment	Support for judgment
	interest	
**Rodriguez-Domínguez M, 2013**		
Methods for selection of participants	Low risk	– **Setting where patients were recruited:** Reported<br>– Quote. "Two STI Units in Madrid, Spain, were involved in the present study. From March 2009 to November 2011 a total of 13,585 samples were collected from 8407 attendees, who came voluntarily for suspected STI as a consequence of sexual risk behavior."<br>– **Selection of MSM with rectal LGV infection:** Reported<br>– There were 81 MSM with rectal LGV infection enrolled in this study. Quote. "They were more often detected in MSM (87/94, 92.5%), but in 2011, seven cases were diagnosed in the heterosexual population (four men and three women". The text and Table 1reported 81 rectal LGV cases were from MSM and one case was from a woman.<br>– **Method for LGV identification:** Reported.<br>– Quote. "The study was based on screening of *C. trachomatis* using commercial kits, followed by real-time *pmpH*-PCR discriminating LGV strains, and finally *ompA* gene was sequenced for phylogenetic reconstruction."
Methods for measuring exposure and outcome variables	Low risk	– **Doxycycline 100 mg twice daily for 21 d was used as treatment:** Reported<br>– Quote. "doxycycline (100 mg/ 12 h for 3 weeks) was prescribed in 75 patients (80%)."<br>– **Method used for repeat test after treatment:** Reported<br>– Quote. "Screening of *C. trachomatis* was based on two molecular commercial tests: Abbott Real Time CT/NG (Abbott Laboratories, Des Plaines, IL, USA) was performed in one STI Unit, and BD-ProbeTec TM ET CT/GC (Becton Dickinson Diagnostic Systems, Sparks, MD, USA) in the second STI Unit…"<br>– **Timing of the test of cure:** Not reported<br>– Quote. "At the end of the treatment protocol 57/91 (63%) treated patients had their disease controlled."
Design-specific sources of bias (excluding confounding)	Not reported	– **Drug compliance:** Not reported<br>– **Sexual re-exposure as potential cause of repeat positivity:** Not reported<br>– **Genotype of positive repeat specimen for discrimination between reinfection and treatment failure:** Not reported<br>– **Antibiotic use shortly before doxycycline or before repeat testing:** Not reported<br>– There were 16 cases that were initially treated with a single dose of azithromycin 1 g but our study did not included these patients in our analysis. From personal communication "there were 54 MSM who received doxycycline alone and underwent a repeat test."
Methods to control confounding	Not reported	– Not reported
Statistical methods	Low risk	– Descriptive analysis used. No sample size calculation.
Conflict of interest	Reported conflict of interest	– Quote. "The authors declare no conflicts of interests."
**Blanco JL, 2015**		
Methods for selection of participants	Low risk	– **Setting where patients were recruited:** Reported<br>– Quote. "A longitudinal prospective study at the STD Unit in the Hospital Clinic was conducted in Barcelona between 6/2010 and 4/2014."<br>– **Selection of MSM with rectal LGV infection:** Reported<br>– Quote. "Adult patients were eligible for inclusion if they presented with clinical manifestation of anorectal syndrome or any clinical suspicion of LGV infection after recent history of unsafe sex…. all MSM…"<br>– **Method for LGV identification:** Reported.<br>– Quote. "…assessed weekly by real-time multiplex polymerase chain reaction (M-PCR) that includes LGV"
Methods for measuring exposure and outcome variables	Low risk	– **Doxycycline 100 mg twice daily for 21 d was used as treatment:** Reported<br>– Quote. " If LGV was detected, at visit 1 (day +7) oral doxycycline 100 mg twice a day for 3 weeks"<br>– **Method used for repeat test after treatment:** Reported

Study, bias	Judgment	Support for judgment
		– Quote. "…real-time multiplex polymerase chain reaction (M-PCR) that includes LGV"<br>– **Timing of the test of cure:** Reported.<br>– Real time multiplex PCR was assessed in patients with doxycycline treatment at the end of treatment (day 21).
Design-specific sources of bias (excluding confounding)	Moderate risk	– **Drug compliance:** Not reported<br>– **Sexual re-exposure as potential cause of repeat positivity:** Not reported<br>– **Genotype of positive repeat specimen for discrimination between reinfection and treatment failure:** Reported<br>– Quote. "…real-time multiplex polymerase chain reaction (M-PCR) that includes LGV"<br>– **Antibiotics use shortly before doxycycline or before repeat testing:** Reported<br>– Quote. "All patients received a single dose of 1 g intramuscular ceftriaxone but were aleatorily assigned to receive, if LGV was detected, at visit 1 (day +7): (i) oral doxycycline 100 mg twice a day for 3 weeks; or (ii) azithromycin 1 g orally once weekly for 3 weeks."
Methods to control confounding	Not reported	– Not reported
Statistical methods	Low risk	– Descriptive analysis used. No sample size calculation.
Conflict of interest	Not reported	– Not reported
